# Defining genetic risk factors for scleroderma-associated interstitial lung disease: *IRF5* and *STAT4* gene variants are associated with scleroderma while *STAT4* is protective against scleroderma-associated interstitial lung disease

**DOI:** 10.1007/s10067-019-04922-6

**Published:** 2020-01-08

**Authors:** Carmel J. W. Stock, Angelo De Lauretis, Dina Visca, Cecile Daccord, Maria Kokosi, Vasilis Kouranos, George Margaritopoulos, Peter M. George, Philip L. Molyneaux, Svetlana Nihtyanova, Felix Chua, Toby M. Maher, Voon Ong, David J. Abraham, Christopher P. Denton, Athol U. Wells, Louise V. Wain, Elisabetta A. Renzoni

**Affiliations:** 1 Interstitial Lung Disease Unit, National Heart and Lung Institute, Imperial College London, Royal Brompton and Harefield NHS Foundation Trust, Sydney Street, London, SW3 6NP UK; 2 Unita’ Operativa Malattie Respiratorie, Ospedale Guido Salvini, Garbagnate Milanese, Milan, Italy; 3 Division of Pulmonary Rehabilitation, Istituti Clinici Scientifici Maugeri, IRCCS, Tradate, Italy; 4 Division of Respiratory Medicine, Lausanne University Hospital (CHUV), Lausanne, Switzerland; 5 Centre for Rheumatology and Connective Tissue Diseases, Royal Free and University College Medical School, London, UK; 6 Department of Health Sciences, University of Leicester, Leicester, UK; 7 National Institute for Health Research, Leicester Respiratory Biomedical Research Centre, Glenfield Hospital, Leicester, UK

**Keywords:** *CD226*, Genetic association, Genetics, *IRAK1*, *IRF5*, SSc-ILD, *STAT4*

## Abstract

Although several genetic associations with scleroderma (SSc) are defined, very little is known on genetic susceptibility to SSc-associated interstitial lung disease (SSc-ILD). A number of common polymorphisms have been associated with SSc-ILD, but most have not been replicated in separate populations. Four SNPs in *IRF5*, and one in each of *STAT4*, *CD226* and *IRAK1*, selected as having been previously the most consistently associated with SSc-ILD, were genotyped in 612 SSc patients, of European descent, of whom 394 had ILD. The control population (*n* = 503) comprised individuals of European descent from the 1000 Genomes Project. After Bonferroni correction, two of the *IRF5* SNPs, rs2004640 (OR (95% CI)1.30 (1.10–1.54), *p*^corr^ = 0.015) and rs10488631 (OR 1.48 (1.14–1.92), *p*^corr^ = 0.022), and the *STAT4* SNP rs7574865 (OR 1.43 (1.18–1.73), *p*^corr^ = 0.0015) were significantly associated with SSc compared with controls. However, none of the SNPs were significantly different between patients with SSc-ILD and controls. Two SNPs in *IRF5*, rs10488631 (OR 1.72 (1.24–2.39), *p*^corr^ = 0.0098), and rs2004640 (OR 1.39 (1.11–1.75), *p*^corr^ = 0.03), showed a significant difference in allele frequency between controls and patients without ILD, as did *STAT4* rs7574865 (OR 1.86 (1.45–2.38), *p*^corr^ = 6.6 × 10^−6^). A significant difference between SSc with and without ILD was only observed for *STAT4* rs7574865, being less frequent in patients with ILD (OR 0.66 (0.51–0.85), *p*^corr^ = 0.0084). In conclusion, *IRF5* rs2004640 and rs10488631, and *STAT4* rs7574865 were significantly associated with SSc as a whole. Only *STAT4* rs7574865 showed a significant difference in allele frequency in SSc-ILD, with the T allele being protective against ILD.

**Table:** 

**Key points** • *We confirm the associations of the IRF5 SNPs rs2004640 and rs10488631, and the STAT4 SNP rs7574865, with SSc as a whole.* • *None of the tested SNPs were risk factors for SSc-ILD specifically.* • *The STAT4 rs7574865 T allele was protective against the development of lung fibrosis in SSc patients.* • *Further work is required to understand the genetic basis of lung fibrosis in association with scleroderma.*

**Electronic supplementary material:**

The online version of this article (10.1007/s10067-019-04922-6) contains supplementary material, which is available to authorized users.

## Introduction

Scleroderma (SSc) is a chronic connective tissue disease characterised by fibrosis of the skin and internal organs, vascular damage and immune dysregulation [1]. SSc is characterised by marked heterogeneity of clinical manifestations and disease course, and its pathogenesis remains poorly understood. SSc carries one of the highest mortality rates among connective tissue diseases, with interstitial lung disease (ILD) being the leading cause of death [2]. Although the majority of SSc-ILD patients have a relatively mild and/or stable lung disease, a substantial minority have progressive lung fibrosis [2]. The molecular pathways which underlie development and progression of SSc-ILD are currently unknown, but are likely to be driven by an interaction between predisposing genetic factors and environmental triggers. Identification of the genetic determinants of lung fibrosis in SSc could improve understanding of pivotal molecular pathways, potentially leading to better prognostic and therapeutic tools for SSc-ILD.

Evidence for a genetic predisposition to SSc as a whole includes a higher prevalence in first degree relatives, and variation in prevalence among different ethnic groups. Twin studies have revealed a strong genetic influence on antinuclear antibody status, in turn linked with internal organ involvement, with ATA antibodies strongly associated with development of SSc-ILD, and ACA antibodies protective for ILD. A number of genes have been consistently associated with SSc as a whole. Similarly to other autoimmune diseases, there is a strong effect of the HLA (human leukocyte antigen) region, mainly with specific autoantibodies [3]. Immune response–related genes are among the most consistently replicated non-HLA associations, including interferon regulatory factor 5 (*IRF5*) [4, 5], signal transducer and activator of transcription 4 (*STAT4*) [6, 7], and cell receptor CD3ζ (*CD247*) [8].

A smaller number of studies have looked at SSc-ILD specifically, although conflicting evidence is reported, with only a few associations replicated in more than one study [9]. Genetic associations reported as specific to SSc-ILD in more than one cohort, including *IRF5*, *STAT4*, DNAX accessory molecule 1 (*CD226*) and interleukin-1 receptor-associated kinase-1 (*IRAK1*). A number of SNPs in *IRF5* have been associated with SSc-ILD, including in a French [10, 11] and a Han Chinese [12] population. *IRF5* SNP rs4728142 was associated with improved survival in SSc [4]. A SNP in *STAT4*, rs7574865, has also been associated with SSc-ILD in a French [13] and a Han Chinese [7] population. *CD226* SNP rs763361 was significantly associated with SSc-ILD in a meta-analysis study of three European populations, with a trend towards significance when each population was analysed separately [14]. The minor allele of rs1059702 in *IRAK1*, on the X chromosome, results in increased NFκ-B activity. Two studies performing meta-analysis on European populations have been reported. In both studies, which both comprised meta-analysis of three populations, rs1059702 was associated with SSc-ILD [15, 16].

In this study, we focused on genes previously reported as risk factors for SSc-ILD in more than one population, and selected the SNPs which had been most consistently associated [9]. We also sought to determine their association with mortality and ILD progression.

## Materials and methods

### Study populations

DNA samples were collected from consecutive, unrelated SSc patients attending clinics at the Royal Brompton and Royal Free Hospitals, London. The diagnoses were made from well-defined criteria for SSc [17]. Only individuals of European descent were included. The control population (*n* = 503) comprised individuals of European descent from the publicly available 1000 Genomes Project [18].

### Clinical assessment

ILD was defined as the presence of fibrosis on chest imaging (chest X-ray or HRCT) and/or a forced vital capacity (FVC) < 75%. Pulmonary function tests (expressed as percent predicted) from the time of first presentation at the Royal Brompton Hospital were available for 578 patients. As a marker of ILD severity which adjusts for the extent of emphysema, the composite physiological index (CPI) was calculated as CPI = 91.0 − (0.65 × DLCO% predicted) − (0.53 × FVC% predicted) + (0.34 × FEV1% predicted) [19]. Time to decline was quantified using serial pulmonary functional indices starting from first visit. Significant functional deterioration was defined as a decline (quantified as percentage change from baseline) of ≥ 10% in FVC and/or of ≥ 15% in DLCO. To allow for possible response to treatment or spontaneous fluctuations, time to irreversible decline was used, defined as time to first significant change observed on at least two consecutive occasions. Data at a sufficient number of time points was available to calculate time to decline in 374 patients. All-cause mortality was also analysed (*n* = 553).

### Genotyping

**Fig. 1:**
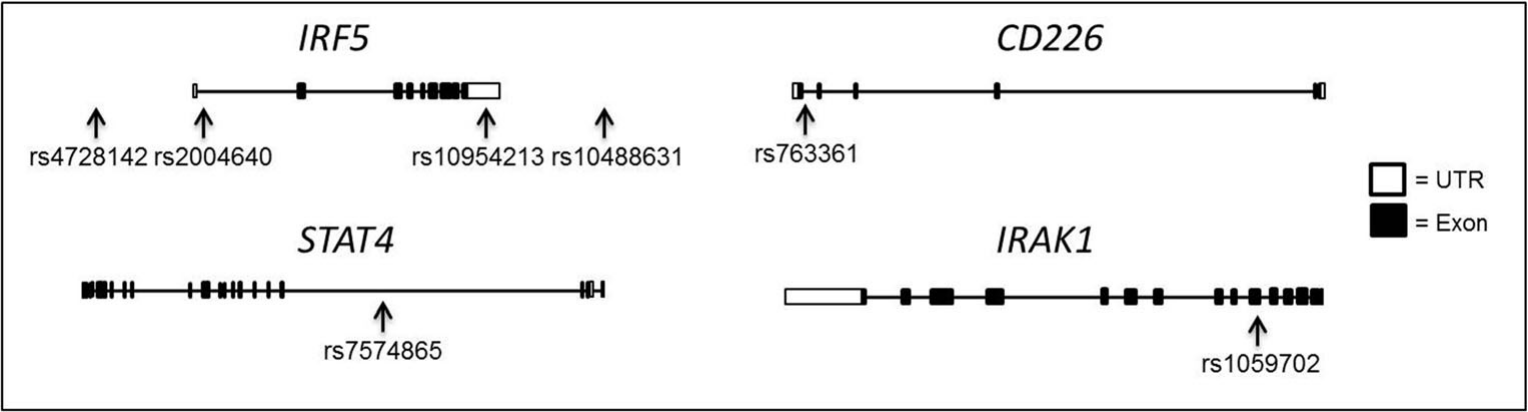
Location of the studied SNPs in *IRF5*, *CD226*, *STAT4* and *IRAK1.* Shown are the locations of the SNPs tested in this study in relation to the gene exons of *IRF5*, *CD226*, S*TAT4* and *IRAK1.* rs4728142 is located in the promoter region of *IRF5* and rs10488631 in the downstream region of *IRF5.* (*IRF5*) Interferon regulatory factor 5, (*CD226*) DNAX accessory molecule 1, (*STAT4*) signal transducer and activator of transcription 4, (*IRAK1*) interleukin-1 receptor-associated kinase-1

The gene locations of the seven SNPs selected for testing are shown in Fig. 1. DNA was extracted from blood using Gentra PureGene DNA kits (Qiagen). Genotyping was carried out according to manufacturer’s instructions using a commercially available TaqMan® assay and TaqMan® universal PCR master mix, no AmpErase® UNG (Applied Biosystems), on a Rotor-Gene 6000 real-time PCR machine (Qiagen). Quality control and genotype determination were performed using the Rotor Gene 6000 Series Software 1.7 (Corbett Research).

### Statistical analysis

To test for deviation from Hardy-Weinberg equilibrium (HWE), genotype frequencies were determined by direct counting, and the chi square statistic or Fisher’s exact test were used as appropriate. Chi square analyses for association were carried out in Unphased v 3.1. To assess the most appropriate genetic model for significant signals, logistic regression analysis was applied in STATA v 15. Only female patients (*n* = 483) and controls (*n* = 263) were included in the analysis of the X chromosome *IRAK1* SNP. Bonferroni correction was applied to correct for multiple testing of seven SNPs. A corrected *p* value (*p*^corr^) < 0.05 was considered significant. The current study had 80% power to detect an association with SSc as a whole with an OR of at least 1.5 for a SNP with a minor allele frequency of 0.5 at *p*^corr^ < 0.05, and 80% power to detect an association with SSc-ILD with an OR of at least 1.6. Cox proportional hazards analysis was used to evaluate time to decline in FVC, time to decline in DLCO, and mortality, as implemented in the Stata v 15.1 (Computing Resource Centre).

## Results

A total of 612 patients were included in the study, of whom 394 had ILD. Patient demographic and clinical characteristics are shown in Table 1. The genotyping success rate for all seven SNPs was ≥ 97.7%. All seven SNPs conformed to Hardy-Weinberg equilibrium in the control population.

**Table 1:** Patient characteristics

Age (range)	52.8 (18.5–93.4)
Gender (female %)	495 (80.9)
Smoking* (never %)	132 (34.9)
Lung function follow-up length (years)	8.2 (0.1–31.8)
Presence of ILD (%)	394 (64.4)
Baseline pulmonary function*	
DLCO% predicted	60.9 (47.9–74.4)
FEV1% predicted	84.7 (71.9–96.0)
FVC% predicted	88.7 (73.9–104.3)
CPI	33.4 (22.9–45.9)
Mortality* (deaths %)	250 (45.2)
Autoantibody* (%)	
ATA	150 (26.5)
ACA	126 (22.3)

Number of patients is equal to 612 unless otherwise stated. Age is age at 1st pulmonary function test/1st hospital visit. Data are presented as age, mean (range), all other data are presented as median (interquartile range), except for follow-up length which is presented as median (range)

*smoking status available for *n* = 378, baseline pulmonary function available for *n* = 583, mortality available for *n* = 553 and autoantibody status available for *n* = 566

*ILD* interstitial lung disease, *DLCO* diffusing capacity of the lung for carbon monoxide, *FEV1* forced expiratory volume in 1 s, *FVC* forced vital capacity, *CPI* composite physiological index, *ATA* anti-topoisomerase antibody, *ACA* anti-centromere antibody

As shown in Table 2, a total of three of the tested SNPs were significantly associated with SSc compared with controls. *IRF5* rs2004640 T allele (OR 1.30 (95% CI 1.10–1.54), *p*^corr^ = 0.015), *IRF5* rs10488631 C allele (OR 1.48 (95% CI 1.14–1.92), *p*^corr^ = 0.022) and *STAT4* rs7574865 T allele (OR 1.43 (95% CI 1.18–1.73), *p*^corr^ = 0.0015) were risk factors for SSc.

**Table 2:** Allele frequency in control, SSc, SSc-ILD and SSc-non ILD cohorts

SNP	Control (*n* = 503)	SSc (*n* = 612)	OR (95% CI)	*p* value	*p*^corr^	SSc-ILD (*n* = 394)	OR (95% CI)	*p* value	*p*^corr^	SSc-no ILD (*n* = 218)	OR (95% CI)	*p* value	*p*^corr^
*IRF5* rs4728142 (G>A)	0.45	0.50	1.21 (1.03–1.44)	0.023	0.16	0.49	1.17 (0.97–1.41)	0.10		0.51	1.30 (1.03–1.62)	0.024	0.17
*IRF5* rs2004640 (G>T)	0.53	0.59	1.30 (1.10–1.54)	0.0022	0.015	0.59	1.25 (1.04–1.51)	0.019	0.13	0.61	1.39 (1.11–1.75)	0.0043	0.03
*IRF5* rs10954213 (G>A)	0.63	0.65	1.08 (0.90–1.28)	0.041	0.29	0.64	1.03 (0.85–1.25)	0.77		0.67	1.16 (0.92–1.47)	0.20	
*IRF5* rs10488631 (T>C)	0.10	0.14	1.48 (1.14–1.92)	0.0031	0.022	0.13	1.34 (1.00–1.80)	0.048	0.34	0.16	1.72 (1.24–2.39)	0.0014	0.0098
*CD226* rs763361 (C>T)	0.47	0.49	1.06 (0.90–1.25)	0.50		0.47	0.98 (0.81–1.18)	0.82		0.52	1.22 (0.97–1.54)	0.081	
*STAT4* rs7574865 (G>T)	0.23	0.30	1.43 (1.18–1.73)	0.00022	0.0015	0.27	1.22 (0.99–1.52)	0.067		0.36	1.86 (1.45–2.38)	0.00000094	0.0000066*
*IRAK1* rs1059702 (G>A)^#^	0.16	0.17	1.12 (0.84–1.49)	0.46		0.16	1.05 (0.76–1.45)	0.78		0.18	1.22 (0.86–1.74)	0.26	

Data are presented as risk allele frequency; *p*^corr^ value is Bonferroni corrected for testing 7 SNPs and is compared with the control cohort

*p^corr^ = 0.0084 SSc no-ILD compared with SSc-ILD

^#^For the X chromosome *IRAK1* SNP, SSc *n* = 483, control *n* = 263, SSc-ILD *n* = 293, SSc-no ILD *n* = 190

*SNP* single nucleotide polymorphism, *SSc* scleroderma, *ILD* interstitial lung disease, *OR* odds ratio, *CI* confidence interval, *IRF5* interferon regulatory factor 5, *CD226* DNAX accessory molecule 1, *STAT4* signal transducer and activator of transcription 4, *IRAK1* interleukin-1 receptor-associated kinase-1

No significant difference in allele frequency of the tested SNPs was observed between patients with SSc-ILD and controls (Table 2). However, the minor allele of two SNPs in *IRF5* were more frequent in SSc patients without ILD than in controls, rs2004640 T allele (OR 1.39 (95% CI 1.11–1.75), *p*^corr^ = 0.03) and rs10488631 C allele (OR 1.72 (95% C 1.24–2.39), *p*^corr^ = 0.0098). *STAT4* SNP rs7574865 T allele was also significantly associated with SSc-non ILD (OR 1.86 (95% CI 1.45–2.38), *p*^corr^ = 6.6 × 10^−6^). *STAT4* rs7574865 T allele was significantly less frequent in SSc patients with ILD than those without (OR 0.66 (95% CI 0.51–0.85), *p*^corr^ = 0.0084) (Table 2). A logistic regression analysis using an additive model provided comparable results (Supplementary table 1).

Given the higher proportion of females in the patient cohort, 80.9% compared with 52.3% in the control population, we performed a logistic regression with sex as a covariate and observed no change to the significance of the association with any of the variants (Supplementary table 2).

None of the seven tested SNPs were associated with mortality (Table 3). An association was seen between *IRF5* rs10488631 and time to decline in FVC by ≥ 10% (OR 1.42 (95% CI 1.08–1.87), *p* = 0.012) and with time to decline in DLCO by ≥ 15% (OR 1.32 (95% CI 1.02–1.71), *p* = 0.038), although neither remained significant when Bonferroni correction was applied (*p*^corr^ = 0.084 and *p*^corr^ = 0.27 respectively). Both of these associations were present on multivariate analysis correcting for age at baseline, gender, smoking status, and disease severity (CPI) (FVC decline by ≥ 10% OR 1.34 (95% CI 1.02–1.85), *p* = 0.04 and DLCO decline by ≥ 15% OR 1.32 (95% CI 1.01–1.74), *p* = 0.044), although again neither remained significant following Bonferroni correction (*p*^corr^ = 0.28 and *p*^corr^ = 0.31, respectively) (Table 3).

**Table 3:** Relationship between individual SNPs, ILD progression and survival

	Univariate			Multivariate*		
SNP	Hazards ratio (CI)	*p* value	*p*^corr^ value	Hazards ratio (CI)	*p* value	*p*^corr^ value
Decline in FVC ≥ 10%						
*IRF5* rs4728142 (G>A)	1.23 (0.86–1.77)	0.25		1.18 (0.81–1.72)	0.38	
*IRF5* rs2004640 (G>T)	0.92 (0.67–1.25)	0.59		0.95 (0.68–1.32)	0.75	
*IRF5* rs10954213 (G>A)	1.25 (0.93–1.69)	0.15		1.16 (0.84–1.61)	0.35	
*IRF5* rs10488631 (T>C)	1.42 (1.08–1.87)	0.012	0.084	1.34 (1.02–1.85)	0.04	0.28
*CD226* rs763361 (C>T)	1.46 (0.99–2.15)	0.056		1.40 (0.94–2.10)	0.10	
*STAT4* rs7574865 (G>T)	0.98 (0.73–1.33)	0.91		1.10 (0.80–1.52)	0.56	
*IRAK1* rs1059702 (G>A)	1.11 (0.79–1.56)	0.55		1.01 (0.71–1.45)	0.94	
Decline in DLCO ≥ 15%						
*IRF5* rs4728142 (G>A)	1.39 (1.00–1.92)	0.05		1.30 (0.93–1.82)	0.13	
*IRF5* rs2004640 (G>T)	0.96 (0.73–1.28)	0.80		0.94 (0.70–1.26)	0.66	
*IRF5* rs10954213 (G>A)	1.49 (0.88–1.52)	0.30		1.15 (0.86–1.52)	0.35	
*IRF5* rs10488631 (T>C)	1.32 (1.02–1.71)	0.038	0.27	1.32 (1.01–1.74)	0.044	0.31
*CD226* rs763361 (C>T)	1.24 (0.89–1.73)	0.20		1.22 (0.87–1.71)	0.26	
*STAT4* rs7574865 (G>T)	0.93 (0.71–1.22)	0.60		1.02 (0.77–1.37)	0.87	
*IRAK1* rs1059702 (G>A)	1.02 (0.75–1.38)	0.91		1.05 (0.77–1.44)	0.76	
Mortality						
*IRF5* rs4728142 (G>A)	0.89 (0.67–1.17)	0.40		0.82 (0.59–1.15)	0.25	
*IRF5* rs2004640 (G>T)	0.96 (0.74–1.24)	0.73		1.06 (0.77–1.46)	0.70	
*IRF5* rs10954213 (G>A)	1.08 (0.83–0.39)	0.57		1.03 (0.75–0.39)	0.87	
*IRF5* rs10488631 (T>C)	0.95 (0.71–1.26)	0.72		0.77 (0.54–1.11)	0.16	
*CD226* rs763361 (C>T)	0.94 (0.70–1.27)	0.70		1.01 (0.70–1.44)	0.98	
*STAT4* rs7574865 (G>T)	0.86 (0.67–1.10)	0.22		1.02 (0.75–1.38)	0.90	
*IRAK1* rs1059702 (G>A)	0.90 (0.67–1.21)	0.50		1.09 (0.76–1.55)	0.64	

*Multivariate analysis correcting for age, gender, smoking history and disease severity (CPI). *p*^corr^ value is Bonferroni corrected for testing 7 SNPs

*SNP* single nucleotide polymorphism, *CI* confidence interval, *FVC* forced vital capacity, *DLCO* diffusing capacity of the lung for carbon monoxide, *IRF5* interferon regulatory factor 5, *CD226* DNAX accessory molecule 1, *STAT4* signal transducer and activator of transcription 4, *IRAK1* interleukin-1 receptor-associated kinase-1

## Discussion

A number of genetic associations with SSc-ILD have been reported. However, conflicting evidence exists, with only a few associations replicated in more than one study [9]. We selected seven SNPs over four genes, for which the most robust evidence of an association with SSc-ILD had been reported, with the aim to test these associations in our UK-based SSc cohort of patients of European descent.

In this study, three of the tested SNPs were significantly associated with SSc as a whole, confirming previous findings [9]. However, we found no evidence that any of the seven SNPs are associated specifically with the presence of ILD. By contrast, we report the novel finding that the *STAT4* rs7574865 T allele may be protective against the development of lung fibrosis in SSc patients.

Although well replicated associations between the *IRF5* and *STAT4* SNPs are reported with SSc as a whole [4–6], conflicting results exist for genetic associations specifically with SSc-ILD. A meta-analysis of five European populations found all three *IRF5* SNPs to be associated with all of the tested SSc subtypes, including, as we found in our study, no ILD [20], suggesting that the *IRF5* association is with SSc as a whole, rather than specifically with ILD. Similarly, although the *STAT4* SNP rs7574865 association with SSc-ILD has been reported in both a French [13] and a Han Chinese [7] population, a study of six European populations found no significant association in any of the populations individually, nor in the meta-analysis [21]. In fact, rs7574865 was associated with limited but not diffuse cutaneous skin disease, the phenotype more frequently associated with ILD [21]. Although a meta-analysis of three European populations found *CD226* SNP rs763361 to be associated with SSc-ILD [14]; a larger study, comprising patients from seven European cohorts, did not confirm an association with the individual SNP, while reporting an association with a haplotype [22]. The *IRAK1* SNP rs1059702, which we did not find to be associated with SSc-ILD nor with SSc as a whole, has been found to be associated with SSc-ILD in two meta-analysis studies of multiple European populations, although not in the individual populations [15, 16].

The observation that the *STAT4* variant is significantly less frequent in patients with SSc-ILD compared with SSc patients without ILD is interesting. However, the findings of this study will need to be replicated in independent populations. No individual SNP is currently sufficiently strongly associated with either SSc or SSc-ILD for use in clinical diagnostics. It is possible that in the future, a panel of genetic variations, possibly combined with other biomarkers, could be utilised in the clinic to aid diagnostics or prognostics, but currently no test is sufficiently powered to provide information for an individual patient.

The current study has some limitations. Even though our SSc-ILD cohort was fairly large for a relatively infrequent entity, being favourably comparable with published single cohort studies reporting an association with SSc-ILD [7, 10, 12], it may have been underpowered to detect small genetic effects. This is particularly true for the *IRAK1* SNP, a gene found on the X chromosome, such that only female patients (*n* = 483) and controls (*n* = 263) were included in this analysis. With these sample sizes, the study had 80% power to detect an association with an OR of at least 1.97 at *p*^corr^ < 0.05, for the *IRAK1* SNP, which has a minor allele frequency of 0.16. SSc-ILD is a complex disease, and it is expected that there will be a number of genetic susceptibility loci, each with modest effect, contributing to increasing the risk of lung fibrosis. We may therefore have been unable to detect associations of small effect size and acknowledge that larger patient sample sizes derived from multicentre collaborations are required to definitively characterise the genetic risk of SSc-ILD. Another limitation is the lack of matching between the patient and the control population. While all patients and controls included in the study were of European descent, we were unable to account for fine-scale population structure as we did not have genome-wide data. Information on age and smoking history of the control population was not available. Although we do not expect age to influence any of the described genetic associations, smoking history could affect our interpretation as we could detect SNP associations with differences in smoking behaviours between cases and controls. As we did not have smoking information for our control population, we have performed a GWAS Catalogue search and have verified that none of the SNPs included in this study are associated with smoking behaviour.

The biological pathways involved in the susceptibility to ILD in patients with SSc remain largely unknown. Immunosuppression has been associated with modest improvement in SSc-ILD [23, 24], suggesting that immune mediated pathways are key drivers of lung fibrosis in this disease, and nintedanib, a multityrosine kinase inhibitor, has recently been approved as treatment for SSc-ILD based upon reduction in decline in lung function in the large SENSCIS clinical trial [25]. The genes investigated in this study are involved in the functioning of the immune system, with most having also been associated with other autoimmune diseases. Whether genes encoding for immune pathways are involved in the genetic predisposition to SSc-ILD uniquely, rather than with SSc as a whole, remains to be determined. On the other hand, the genetic risk may lie within genes involved in aberrant wound healing/pro-fibrotic pathways. A progressive fibrotic phenotype despite immunosuppression is observed in a subset of patients with SSc-ILD, and anti-fibrotic treatments currently used in IPF, have recently shown promise also in SSc-ILD [25]. However, despite some similarities with IPF and other idiopathic interstitial pneumonias (IIPs), SSc-ILD appears to be genetically distinct. Loci associated with IIPs tend to be involved with host-defence, epithelial injury/dysfunction and wound healing [26]. The gain-of-function mucin 5B (*MUC5B*) promoter variant, the most consistent common genetic risk factor for IIPs, is not associated with SSc-ILD [27]. Similarly, other genetic susceptibility loci identified in recent genome-wide association studies in IIPs have not been confirmed in SSc-ILD cohorts [26]. By contrast, IIP associated variants have also been found in rheumatoid arthritis-ILD, and for the *MUC5B* variant at least, specifically with an underlying usual interstitial pneumonia (UIP) pattern [28]. As SSc-ILD is most frequently characterised by a fibrotic non-specific interstitial pneumonia (NSIP) pattern, one can speculate that the genetic architecture may differ between these two histological patterns. However, there may instead be disease-specific genetic risks for SSc-ILD, which will require an approach focused on the development and progression of ILD in SSc patients.

The majority of previous studies have focused on SSc as a whole, with SSc-ILD investigated as a post hoc sub-analysis. These studies are therefore often underpowered to detect associations with SSc-ILD specifically. Furthermore, few have sought out to detect genetic risk factors for significant ILD outcomes, including lung function decline. With the availability of large cohorts with adequate long-term lung function follow-up, it should be possible to detect specific genetic associations with a progressive fibrotic phenotype and/or potential gene variants associated with increased likelihood of response to anti-inflammatory or anti-fibrotic agents. This study highlights the need for more, adequately powered, studies addressing the specific question of the genetic susceptibility to SSc-ILD. This will require international collaborations aimed at performing hypothesis-free genome-wide association studies specifically targeted at well-defined SSc-ILD cross-sectionally and longitudinally.

## Electronic supplementary material

ESM 1 (DOCX 22 kb)
