# Supplementary material for: Defining genetic risk factors for scleroderma-associated interstitial lung disease: IRF5 and STAT4 gene variants are associated with scleroderma while STAT4 is protective against scleroderma-associated interstitial lung disease
Source: Clin Rheumatol. 2020 Jan 8;39(4):1173–9. doi: 10.1007/s10067-019-04922-6 (PMC7142048; doi:10.1007/s10067-019-04922-6)
Supplement: Supplementary file 1 — (DOCX 22 kb) [file 10067_2019_4922_MOESM1_ESM.docx]

**Supplementary Table 1. Genotype frequencies in control, SSc, SSc-ILD, and SSc-non ILD cohorts**

| **SNP** | **Control**  **(n=503)** | **SSc**  **(n=612)** | **OR (95% CI)** | **p value** | **p^corr^** | **SSc-ILD**  **(n=394)** | **OR (95% CI)** | **p value** | **p^corr^** | **SSc-no ILD**  **(n=218)** | **OR (95% CI)** | **p value** | **p^corr^** |
| --- | --- | --- | --- | --- | --- | --- | --- | --- | --- | --- | --- | --- | --- |
| *IRF5* rs4728142 (G>A) | GG:0.31  GA:0.49  AA:0.20 | GG:0.25  GA:0.51  AA:0.24 | 1.21 (1.02-1.43) | 0.026 | 0.18 | GG:0.26  GA:0.50  AA:0.24 | 1.18 (0.98-1.42) | 0.09 |  | GG:0.23  GA:0.51  AA:0.26 | 1.29 (1.04-1.61) | 0.023 | 0.061 |
| *IRF5* rs2004640 (G>T) | GG:0.23  GT:0.48  TT:0.29 | GG:0.16  GT:0.48  TT:0.36 | 1.29 (1.09-1.53) | 0.003 | 0.021 | GG:0.16  GT:0.50  TT:0.34 | 1.24 (1.03-1.50) | 0.023 | 0.16 | GG:0.16  GT:0.45  TT:0.39 | 1.41 (1.13-1.76) | 0.002 | 0.014 |
| *IRF5* rs10954213 (G>A) | GG:0.14  AG:0.46  AA:0.40 | GG:0.13  AG:0.45  AA:0.42 | 1.08 (0.90-1.28) | 0.43 |  | GG:0.13  AG:0.46  AA:0.41 | 1.03 (0.85-1.25) | 0.73 |  | GG:0.11  AG:0.45  AA:0.44 | 1.16 (0.93-1.47) | 0.19 |  |
| *IRF5* rs10488631 (T>C) | TT:0.81  TC:0.18  CC:0.01 | TT:0.74  TC:0.23  CC:0.03 | 1.45 (1.12-1.88) | 0.004 | 0.028 | TT:0.77  TC:0.20  CC:0.03 | 1.33 (0.99-1.77) | 0.053 |  | TT:0.70  TC:0.28  CC:0.02 | 1.61 (1.16-2.23) | 0.004 | 0.028 |
| *CD226* rs763361 (C>T) | CC:0.27  CT:0.51  TT:0.22 | CC:0.25  CT:0.52  TT:0.23 | 1.06 (0.89-1.25) | 0.52 |  | CC:0.27  CT:0.52  TT:0.21 | 0.98 (0.81-1.19) | 0.86 |  | CC:0.21  CT:0.53  TT:0.26 | 1.16 (0.93-1.45) | 0.20 |  |
| *STAT4* rs7574865 (G>T) | GG:0.60  GT:0.34  TT:0.06 | GG:0.51 GT:0.38  TT:0.11 | 1.41 (1.17-01.69) | <0.001 | <0.007 | GG:0.55  GT:0.37  TT:0.08 | 1.21 (0.98-1.49) | 0.084 |  | GG:0.43  GT:0.42  TT:0.15 | 1.73 (1.37-2.20) | <0.001 | <0.007* |
| *IRAK1* rs1059702 (G>A) | GG:0.70  GA:0.29  AA:0.01 | GG:0.69  GA:0.28  AA:0.03 | 1.12 (0.84-1.50) | 0.45 |  | GG:0.71  GA:0.26  AA:0.03 | 1.04 (0.75-0.91) | 0.81 |  | GG:0.66  GA:0.31  AA:0.03 | 1.24 (0.87-1.76) | 0.23 |  |

Data are presented as genotype frequency. Data were analysed using logistic regression (additive model). p^corr^ value is Bonferroni corrected for testing 7 SNPs and is compared to the control cohort. * p^corr^=0.014 SSc no-ILD compared to SSc-ILD. Definition of abbreviations: SNP: single nucleotide polymorphism, SSc: Scleroderma, ILD: interstitial lung disease, OR: odds ratio, CI: confidence interval, *IRF5*: interferon regulatory factor 5, *CD226*: DNAX accessory molecule 1, *STAT4*: signal transducer and activator of transcription 4, *IRAK1*: interleukin-1 receptor-associated kinase-1.

**Supplementary Table 2. Genotype frequencies in control, SSc, SSc-ILD, and SSc-non ILD cohorts, including sex as a covariate**

| **SNP** | **Control**  **(n=503)** | **SSc**  **(n=612)** | **OR (95% CI)** | **p value** | **p^corr^** | **SSc-ILD**  **(n=394)** | **OR (95% CI)** | **p value** | **p^corr^** | **SSc-no ILD**  **(n=218)** | **OR (95% CI)** | **p value** | **p^corr^** |
| --- | --- | --- | --- | --- | --- | --- | --- | --- | --- | --- | --- | --- | --- |
| *IRF5* rs4728142 (G>A) | GG:0.31  GA:0.49  AA:0.20 | GG:0.25  GA:0.51  AA:0.24 | 1.23 (1.03-1.46) | 0.022 | 0.15 | GG:0.26  GA:0.50  AA:0.24 | 1.20 (0.99-1.45) | 0.067 |  | GG:0.23  GA:0.51  AA:0.26 | 1.31 (1.04-1.64) | 0.021 | 0.15 |
| *IRF5* rs2004640 (G>T) | GG:0.23  GT:0.48  TT:0.29 | GG:0.16  GT:0.48  TT:0.36 | 1.34 (1.13-1.60) | 0.001 | 0.007 | GG:0.16  GT:0.50  TT:0.34 | 1.29 (1.06-1.57) | 0.01 | 0.07 | GG:0.16  GT:0.45  TT:0.39 | 1.45 (1.15-1.82) | 0.001 | 0.007 |
| *IRF5* rs10954213 (G>A) | GG:0.14  AG:0.46  AA:0.40 | GG:0.13  AG:0.45  AA:0.42 | 1.11 (0.93-1.32) | 0.27 |  | GG:0.13  AG:0.46  AA:0.41 | 1.05 (0.87-1.28) | 0.60 |  | GG:0.11  AG:0.45  AA:0.44 | 1.23 (0.97-1.56) | 0.086 |  |
| *IRF5* rs10488631 (T>C) | TT:0.81  TC:0.18  CC:0.01 | TT:0.74  TC:0.23  CC:0.03 | 1.44 (1.10-1.89) | 0.007 | 0.049 | TT:0.77  TC:0.20  CC:0.03 | 1.31 (0.98-1.76) | 0.07 |  | TT:0.70  TC:0.28  CC:0.02 | 1.68 (1.19-2.38) | 0.003 | 0.021 |
| *CD226* rs763361 (C>T) | CC:0.27  CT:0.51  TT:0.22 | CC:0.25  CT:0.52  TT:0.23 | 1.04 (0.87-1.25) | 0.64 |  | CC:0.27  CT:0.52  TT:0.21 | 0.98 (0.80-1.19) | 0.81 |  | CC:0.21  CT:0.53  TT:0.26 | 1.14 (0.90-1.44) | 0.29 |  |
| *STAT4* rs7574865 (G>T) | GG:0.60  GT:0.34  TT:0.06 | GG:0.51 GT:0.38  TT:0.11 | 1.39 (1.15-1.69) | 0.001 | 0.007 | GG:0.55  GT:0.37  TT:0.08 | 1.19 (0.96-1.48) | 0.12 |  | GG:0.43  GT:0.42  TT:0.15 | 1.76 (1.36-2.26) | <0.001 | <0.007* |

Data are presented as genotype frequency. Data were analysed using logistic regression (additive model). p^corr^ value is Bonferroni corrected for testing 7 SNPs and is compared to the control cohort. * p^corr^=0.014 SSc no-ILD compared to SSc-ILD. Definition of abbreviations: SNP: single nucleotide polymorphism, SSc: Scleroderma, ILD: interstitial lung disease, OR: odds ratio, CI: confidence interval, *IRF5*: interferon regulatory factor 5, *CD226*: DNAX accessory molecule 1, *STAT4*: signal transducer and activator of transcription 4, *IRAK1*: interleukin-1 receptor-associated kinase-1.
